# Beyond grip strength: selective slowing of grip force release but not initiation dynamics in a go/no-go target force–matching task

**DOI:** 10.3389/fnagi.2026.1784018

**Published:** 2026-06-25

**Authors:** Syed Qadri, Jose Roberto Torres Andrade, Seraphina Culp, Beatrice Lee, Emre Umucu, Peter S. Lum, Shashwati Geed

**Affiliations:** 1 Jefferson Moss-Magee Rehabilitation, Elkins Park, PA, United States; 2 Interdisciplinary Health Sciences PhD Program, The University of Texas at El Paso, El Paso, TX, United States; 3 Department of Biomedical Engineering, The Catholic University of America, Washington, DC, United States; 4 Department of Rehabilitation Sciences, The University of Texas at El Paso, El Paso, TX, United States; 5 Department of Public Health Sciences, College of Health Sciences, The University of Texas at El Paso, El Paso, TX, United States; 6 Institute for Health and Lifespan Research (IHLR), Research & Innovation, The University of Texas at El Paso, El Paso, TX, United States; 7 Audie L. Murphy Memorial VA Hospital, South Texas VA Medical Center, San Antonio, TX, United States; 8 Director of CHS REACHED, College of Health Sciences, The University of Texas at El Paso, El Paso, TX, United States; 9 Department of Physical Therapy and Movement Sciences, The University of Texas at El Paso, El Paso, TX, United States

**Keywords:** aging, go/no-go paradigm, motor inhibition, prehension, psychomotor performance, reaction time

## Abstract

**Introduction:**

Precise regulation of grip forces is fundamental for skilled object manipulation and independent navigation of everyday activities throughout the lifespan. Maximal grip strength is a widely used biomarker of aging and frailty, but it provides limited insight into the temporal and inhibitory control mechanisms required for dexterous prehension. Evidence suggests that grip-force release, which requires integration of sensory feedback and inhibition of sustained grip, may be particularly vulnerable to aging.

**Methods:**

Thirty‑six right‑handed younger and older adults performed a go/no-go target force–matching task in which they generated and maintained a submaximal grip in response to a go cue and withheld responses to a no-go cue. The paradigm was designed to separately quantify the timing and execution dynamics of grip onset and release within a continuous force‑control task, yielding measures of onset and offset reaction times (RTs), force rise and release durations, and commission errors on no‑go trials.

**Results:**

Compared with younger adults, older adults exhibited significantly longer grip‑offset RTs but preserved grip‑onset RTs, indicating selective slowing of release initiation. Grip‑force release duration was also significantly longer in older adults, whereas grip‑force rise times did not differ between groups. Older adults committed more commission errors on no‑go trials; error‑onset RTs were comparable across groups, whereas error force‑release duration was prolonged in older adults.

**Discussion:**

These findings indicate that aging selectively slows the initiation and execution of grip release, in a manner consistent with vulnerabilities in inhibitory processes rather than generalized motor slowing. The results extend prior evidence of release‑phase vulnerability in aging by separately quantifying release initiation and execution within a paradigm that also indexes inhibitory control failure, and suggest that grip‑offset dynamics may complement traditional grip‑strength assessments in characterizing age‑related motor decline.

## 1 Introduction

Precise grip force regulation underlies everyday manual dexterity, allowing individuals to secure, manipulate, and release objects safely. While grip strength is often used as a biomarker for aging and frailty (Bohannon, 2019), such global measures do not completely capture the spatiotemporal kinematics and dynamics of grasping, particularly the speeds with which grasp is initiated and terminated. Subtle delays in initiating or terminating grip forces, or failures to suppress inappropriate responses increase the risk of mishandling objects in older adults with direct consequences on activity and participation in independent living (McKniff et al., 2025).

Aging is associated with declines in several domains relevant to grip regulation (Zammit et al., 2019), including slower reaction times, reduced tactile sensitivity, and diminished inhibitory control (Ruitenberg et al., 2019). However, these changes do not appear to affect all phases of movement equally. Behavioral studies of precision grip tasks have shown that older adults are disproportionately impaired during the release phase (Davidson et al., 2024), when force must be rapidly terminated, and that these deficits are linked to sensory decline (Lin et al., 2019; Voelcker-Rehage and Alberts, 2005; Vieluf et al., 2015; Reuter et al., 2014). Consistent with these findings (Lin et al., 2021), demonstrated that grip force stability control is compromised in older adults during dual-task conditions requiring simultaneous arm reaching, with older adults showing reduced grip strength and increased force variability relative to a target force compared with younger adults. Neuroimaging work further supports the idea that grip release is not simply the inverse of grip initiation: release recruits additional prefrontal and cingulate regions, consistent with the engagement of inhibitory control mechanisms (Spraker et al., 2009). Age-related decline in parietal-motor cortical connectivity may further compound these force control deficits: dual-site transcranial magnetic stimulation (TMS) evidence indicates that facilitator inputs from posterior parietal cortex to ipsilateral primary motor cortex are absent in older adults, and that this reduction is associated with declining hand dexterity, implicating disrupted sensorimotor integration as a contributor to age-related impairment in motor transitions requiring online feedback (Goldenkoff et al., 2021). Taken together, this evidence suggests that grip release is a distinct and particularly vulnerable component of motor control in aging, shaped by both sensory degradation and reduced inhibitory capacity.

Beyond force initiation and termination, effective object handling also requires the ability to withhold inappropriate responses. Go/no-go paradigms consistently demonstrate that older adults commit more commission errors than younger adults, reflecting impaired inhibitory control (Divers et al., 2021). However, it remains unclear whether these inhibitory failures also alter the *timing* and *execution dynamics* of erroneous responses, or whether they simply increase the frequency of errors. Recent evidence suggests that age-related slowing may be most pronounced at the level of movement preparation rather than initiation or execution per se (Hardwick et al., 2022), raising the possibility that erroneous responses, once triggered, may be executed with comparable timing in younger and older adults.

A limitation of prior research is that conventional paradigms investigating aging-associated motor control and reaction times do not separately quantify the timing of response initiation from the continuous dynamics of force production and relaxation. Standard go/no-go tasks using discrete button presses collapse response selection and motor execution into a single latency measure, making it difficult to determine whether age-related slowing reflects delayed response selection, impaired force generation, or slowed disengagement from an ongoing motor state. To address this gap, we implemented a go/no-go target force matching paradigm that separately measures the reaction times associated with grip onset and offset alongside the corresponding durations of force rise and release within the same continuous-force task. This design enabled independent characterization of the timing of response initiation (grip onset and offset reaction times) and the dynamics of force execution (rise and release durations), without asserting that cognitive and motor processes are fully separable.

We hypothesized that (1) grip-offset reaction times and force release durations would be disproportionately prolonged in older adults relative to younger adults, consistent with prior evidence for selective vulnerability of grip-force termination with aging; (2) that grip-onset reaction times and force rise durations would be relatively preserved cross age groups, arguing against a generalized decline in sensorimotor speed; and (3) older adults would commit more commission errors on no-go trials than younger adults, reflecting age-related decline in inhibitory control, but that error onset reaction times would not differ between groups once an erroneous response was initiated – a pattern that, if confirmed, would suggest that aging selectively affects the termination rather than the triggering of force response, regardless of whether those responses are intentional or erroneous.

## 2 Methods

Thirty-six right-handed, community-dwelling adults participated in the study. Participants were excluded if they reported a history of neurological disorders including dementia, Alzheimer’s disease, traumatic brain injury, or stroke, or musculoskeletal conditions that could interfere with task performance. Participants in the younger adult group were aged 18–40 years, whereas participants in the older adult group were aged 60 years or older at the time of consent; individuals aged 41–59 were excluded *a priori* to ensure clear age separation between groups. All participants had normal or corrected-to-normal vision. The study protocol was approved by the MedStar Health Research Institute Institutional Review Board, and all participants provided written informed consent prior to participation.

### 2.1 Experimental setup

Participants were seated in a flat-back chair with the tested arm supported comfortably on a table. A computer monitor positioned 24 inches from the participant at eye level displayed the task cues and visual feedback. Grip force was recorded using an E-LINK Hand Grip Strength Dynamometer (Biometrics Ltd., Newport, UK). The output of the dynamometer controlled a vertical force bar on the screen allowing participants to visually match their grip force to 5% of age- and sex-normed maximum grip force target force levels (Massy-Westropp et al., 2011). A target force of 5% age- and sex-normed maximal grip force was selected to approximate the submaximal grip forces characteristics of everyday prehension and object release, thereby enhancing the functional relevance of the task while ensuring the task was achievable across participants of varying age and grip capacity (Voelcker-Rehage and Alberts, 2005; Massy-Westropp et al., 2011).

### 2.2 Task design

**Figure 1:**
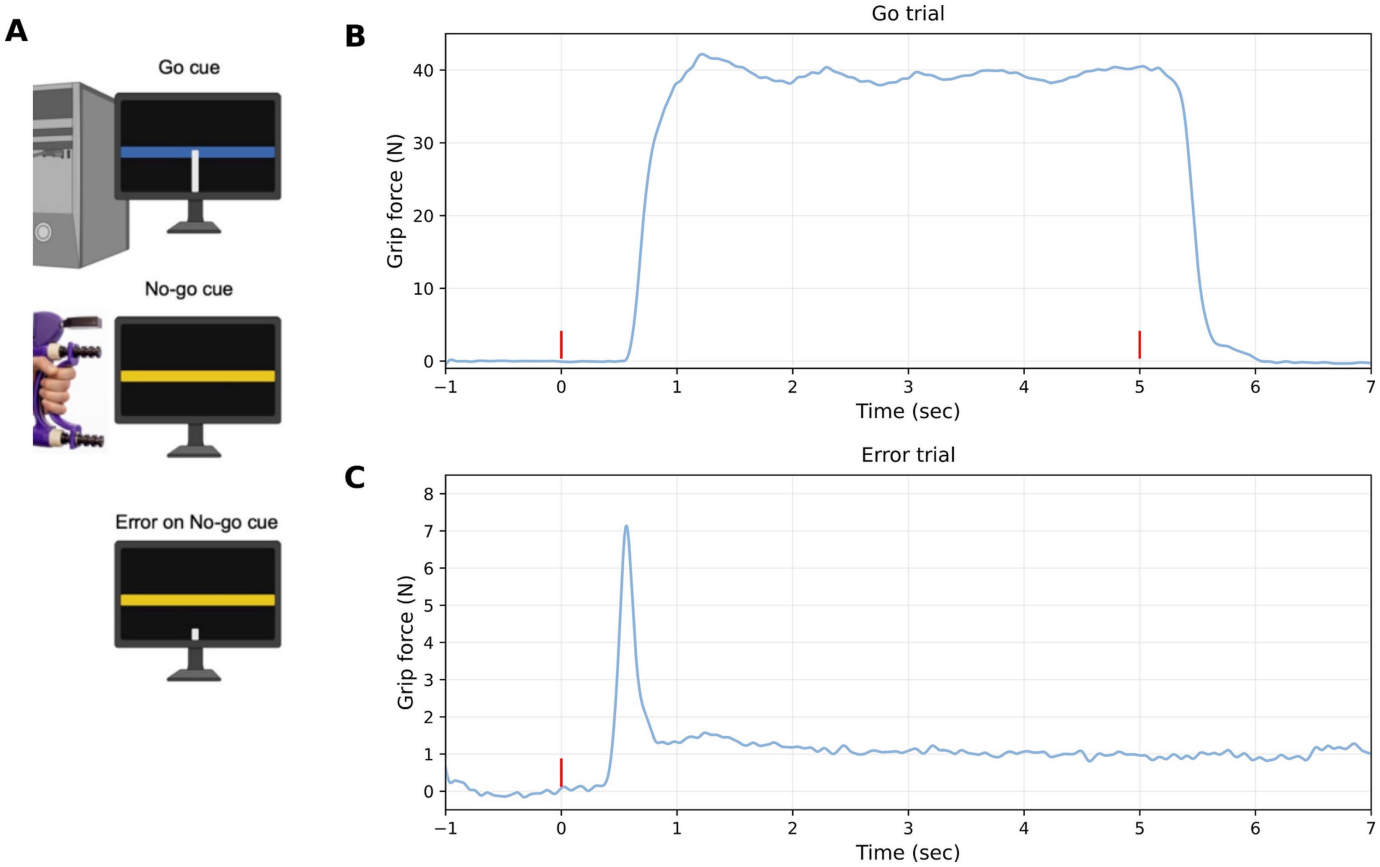
Behavioral task and representative grip force profiles for go, no-go, and error trials. **(A)** Schematic of the go/no-go target force–matching task. Participants viewed a narrow horizontal bar presented on a monitor. A blue bar indicated a go trial, instructing participants to generate grip force and maintain it within the target boundaries. A yellow bar indicated a no-go trial, instructing participants to withhold any response. **(B)** Representative grip force trace (N) during a go trial, illustrating force onset, maintenance at the 5% MVC target, and force release following cue offset. **(C)** Representative grip force trace (N) during a commission error on a no-go trial, illustrating unintended force generation in response to a no-go cue.

Participants completed 80 randomized go/no-go trials presented in repeating 10-trial blocks with a total of 40-go and 40-no-go trials. Each trial displayed a horizontal cue bar on the monitor (Figure 1A). During Go trials (blue bar) participants were instructed to rapidly squeeze the dynamometer to match a prescribed target force and maintain their force within the displayed boundaries until cue offset. During no-go trials (yellow bar), participants were instructed to withhold any grip response.

Cue offset was indicated by the disappearance of the bar, at which point participants were instructed to release their grip immediately. This design enabled measurement of both grip onset and offset dynamics within each go trial (Figure 1B). Target force levels were individualized using age- and sex-normed reference values to ensure comparable task difficulty across participants (Massy-Westropp et al., 2011). All participants completed several practice trials prior to data collection to become familiar with the device and task demands.

### 2.3 Data processing

Grip force signals were processed using custom scripts in MATLAB R2021a (MathWorks, Natick, MA). Raw force signals were low-pass filtered at 10 Hz to attenuate high-frequency noise and subsequently differentiated to compute grip force rate, which facilitates precise identification of grip force onset and release events.

**Figure 2:**
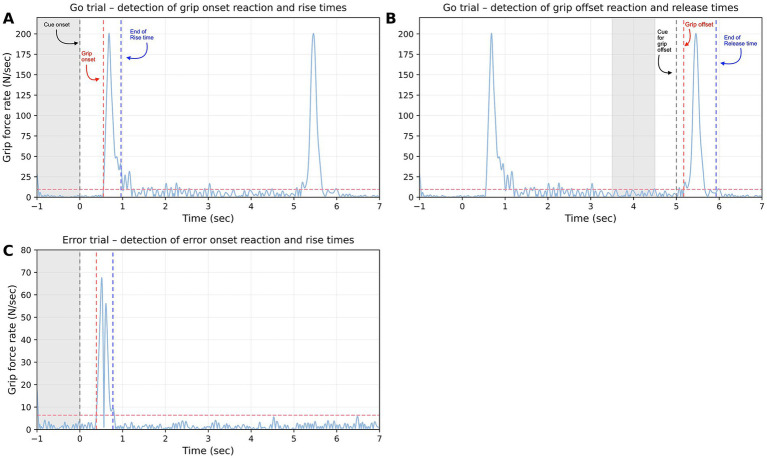
Detection of reaction times and execution durations for grip onset and release. Grip force rate (first derivative of force, N/s) traces are shown for representative trials to illustrate event detection procedures. Gray shaded regions denote the baseline rest period used to compute mean and standard deviation of force rate. Dashed horizontal lines indicate ±2 SD thresholds. Dashed vertical gray line indicates the cue onset at time 0 s and cue offset at time 5-s. **(A)** Go trial illustrating detection of grip onset reaction time (interval between dashed vertical lines in gray and red) and grip force rise duration (interval between dashed vertical lines in red and blue), defined by sustained threshold crossing of force rate. **(B)** Go trial illustrating detection of grip offset reaction time (interval between dashed vertical lines in gray and red) and grip force release duration (interval between dashed vertical lines in red and blue) following cue offset at 5 s. **(C)** Commission error on a no-go trial illustrating detection of error onset reaction time and force rise duration. Event onsets were defined as threshold crossings sustained for at least 80 msec.

Grip onset and offset were detected using a ± 2 standard deviation (SD) threshold applied to the grip force rate signal, calculated from a baseline rest period preceding cue onset (Figure 2). Standard deviation-based thresholds are widely used in physiological signal detection because they scale with baseline variability and provide a data-driven criterion for distinguishing meaningful changes in force rate from background fluctuations (Pinto and Callaghan, 2023; Raez et al., 2006). To avoid classifying transient noise as genuine events, threshold crossings were required to be sustained for at least 80 ms. Minimum duration constraints of this magnitude are commonly applied in onset/offset detection due to the brief nature of mechanical and sensor noise transients (<50-80 ms) and because voluntary force responses typically have latencies exceeding 120-150 ms (Carvalho et al., 2023). This criterion therefore excludes short-lived artifacts without delaying true event identification. All trials were visually inspected for gross signal artifacts; all participants contributed complete datasets, and no trials were excluded from analyses.

From detected events, the following temporal measures were extracted: (1) Onset reaction time: time from cue appearance to the initiation of force increase; (2) Rise duration: time from onset to stable attainment of the target force; (3) Offset reaction time: time from cue disappearance to the initiation of force release; (4) Release duration: time from release initiation to return to baseline force. Figures 2A,B illustrate the criteria used to compute grip force reaction times, grip force rise duration, and grip force release duration.

No-go/error processing: a commission error on a no-go trial (Figure 1C) was defined as force exceeding the onset threshold within a 2-s window following the appearance of the cue (yellow bar). This criterion ensures exclusion of spurious fluctuations while capturing volitional responses that reflect inhibitory control failures. For each error trial, onset and offset metrics were computed using the same procedures as for go trials, enabling characterization of both the frequency of commission errors and their associated force dynamics. Figure 2C illustrates the criteria used to detect commission errors and quantify error onset and execution dynamics during no-go trials.

In summary, the following primary dependent variables were analyzed: for Go trials: (1) Grip onset reaction time; (2) Grip-force rise duration; (3) Grip-offset reaction time; (4) Grip-force release duration. For No-go trials: (6) Number of commission errors; (7) Error-onset reaction time; (8) Error force-release duration.

### 2.4 Statistical analysis

Group differences in continuous outcome measures (reaction times and rise, release durations) were assessed using Welch’s independent-samples-t-tests, which do not assume homogeneity of variance. To control for multiple comparisons, Bonferroni-corrected significance thresholds were applied within each family of analyses (alpha = 0.0125 for Go trial outcomes; alpha = 0.025 for No-go/error trial outcomes). Group differences in the number of commission errors were assessed using a Wilcoxon rank-sum test due to the count-based and non-normality distributed nature of the data. The use of variance-robust and nonparametric tests was motivated by observed heterogeneity of variance and non-normal distributions in select outcome measures. Effect size for each Welch’s t-test comparison was quantified using Hedge’s g, computed using the unweighted pooled standard deviation, which provides a less biased estimate than Cohen’s d for samples of this size. For the Wilcoxon rank-sum test, effect size was expressed as rank-biserial correlation r. All statistical analyses were performed using JMP Pro 19 (SAS Institute Inc., Cary, NC).

## 3 Results

### 3.1 Participant characteristics

**Figure 3:**
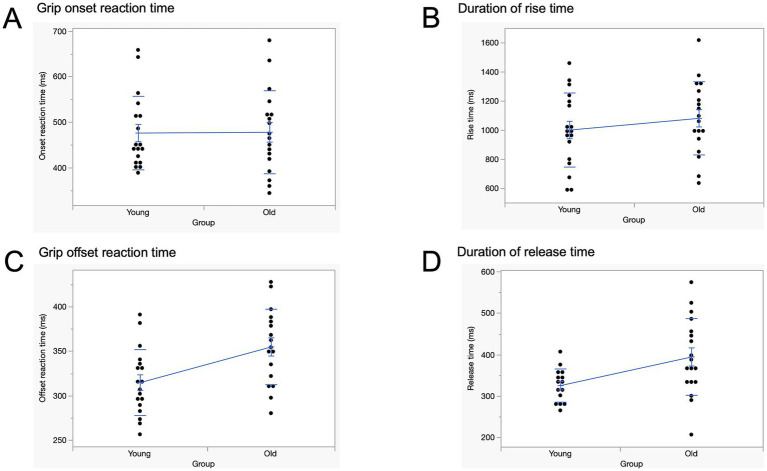
Go-trial grip force timing and execution dynamics. **(A)** Grip onset reaction time. **(B)** Grip force rise duration. **(C)** Grip offset reaction time. **(D)** Grip force release duration. Group means ± standard error of the mean (SEM) are shown, with horizontal lines indicating group standard deviations. Group differences were assessed using Welch’s independent-samples t-tests with Bonferroni-corrected significance thresholds (*α* = 0.0125).

**Figure 4:**
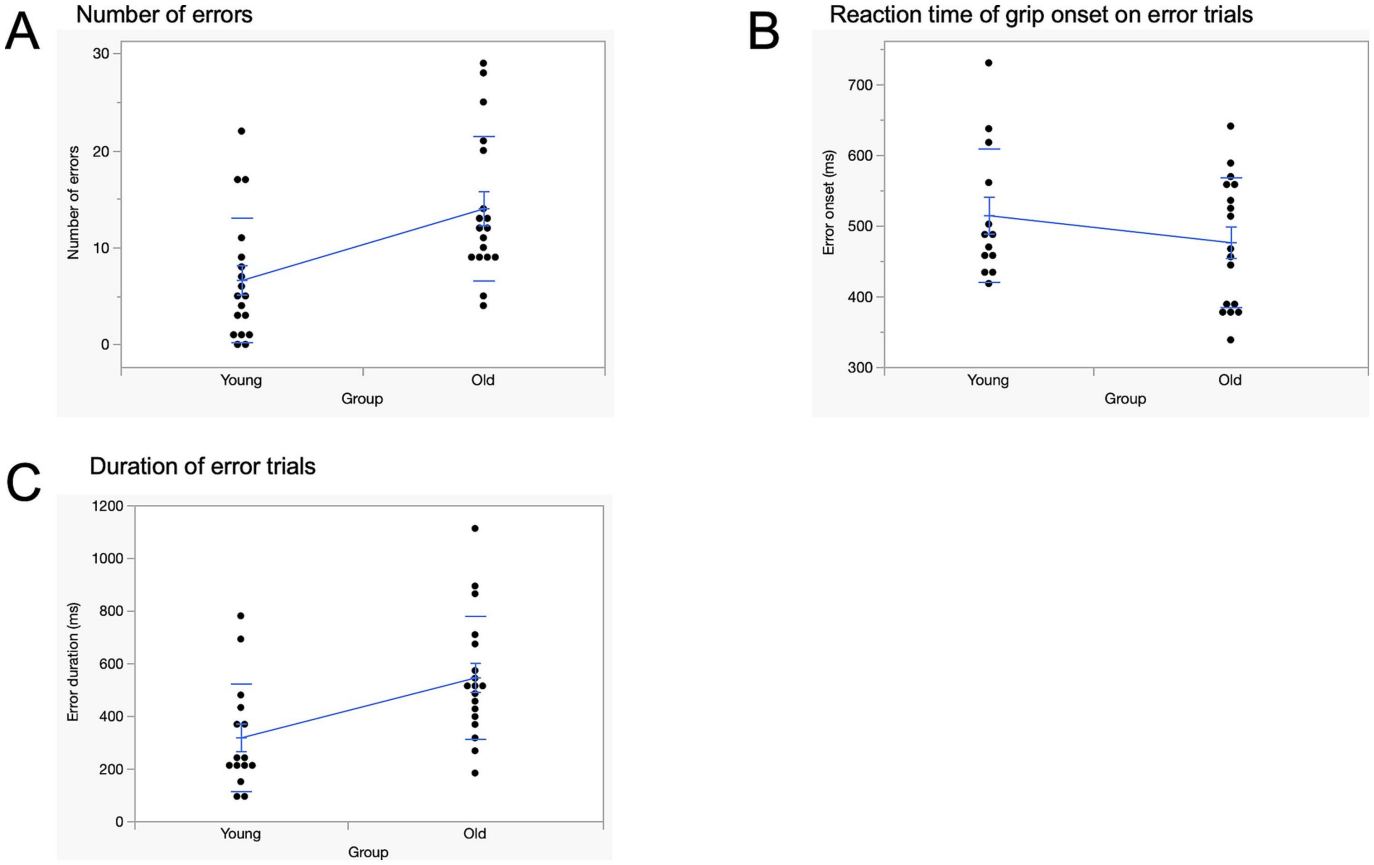
No-go trial performance and error-related force dynamics. **(A)** Number of commission errors during no-go trials for younger and older adults. Group differences were assessed using a Wilcoxon rank-sum (Mann–Whitney U) test. **(B)** Error onset reaction time during commission errors on no-go trials. **(C)** Error force release duration (grip offset execution) during commission errors. Group means ± standard error of the mean (SEM) are shown, with horizontal lines indicating group standard deviations. For continuous error-related measures, group differences were assessed using Welch’s independent-samples t-tests with Bonferroni-corrected significance thresholds (α = 0.025).

Thirty-six adults participated in the study. The younger adult group consisted of 18 participants (16 female, 2 male) with a mean age of 26.9 ± 6.1 years (range: 20–40 years). The older adult group consisted of 18 participants (15 female, 3 male) with a mean age of 75.8 ± 7.5 years (range: 62–93 years). All participants completed the experimental protocol. Figure 3 illustrates the go-trial grip force timing and execution measures, and Figure 4 illustrates no-go trial performance and error-related force dynamics.

### 3.2 Go-trial grip force dynamics

Grip onset reaction time did not differ significantly between age groups. Younger adults exhibited a mean onset reaction time of 476.3 ± 80.8 ms whereas older adults exhibited a mean onset reaction time of 478.1 ± 91.2 ms (Welch’s t = 0.06, *p* = 0.95, Hedge’s g = 0.02, 95% CI [−0.64, 0.68]). Similarly, grip force rise duration did not differ between groups, with younger adults requiring 1000.6 ± 255.3 ms and older adults requiring 1081.4 ± 252.1 ms to reach the target force (Welch’s *t* = 0.95, *p* = 0.35, Hedge’s *g* = 0.31, 95% CI [−0.35, 0.97]).

In contrast, grip offset reaction time was significantly prolonged in older adults compared with younger adults. Younger adults exhibited a mean grip offset reaction time of 314.8 ± 37.2 ms, whereas older adults exhibited a mean grip offset reaction time of 355.1 ± 42.5 ms (Welch’s *t* = 2.97, *p* = 0.0056, Hedge’s g = 0.99, 95% CI [0.31, 1.67]), which remained significant after Bonferroni correction (alpha = 0.0125). Grip force release duration was also significantly longer in older adults (394.9 ± 92.6 ms) than in younger adults (325.9 ± 40.1 ms; Welch’s *t* = 2.86, *p* = 0.0087, Hedge’s g = 0.95, 95% CI [0.27, 1.62]), surviving Bonferroni correction.

### 3.3 No-go trials and error-trial dynamics

Older adults committed significantly more commission errors during no-go trials than younger adults. Over 40 no-go trials, younger adults committed a mean of 6.7 ± 6.4 commission errors, whereas older adults committed a mean of 14.1 ± 7.4 commission errors. This group difference was significant (Wilcoxon rank-sum test, *Z* = 3.16, *p* = 0.0016, rank-biserial r = 0.53).

For commission error trials, error onset reaction time did not differ between age groups. Younger adults exhibited a mean error onset reaction time of 514.9 ± 94.3 ms, whereas older adults exhibited a mean error onset reaction time of 476.6 ± 91.8 ms (Welch’s *t* = 1.11, *p* = 0.28, Hedge’s g = 0.41, 95% CI [−0.29, 1.10]). In contrast, error force execution differed between groups: older adults exhibited significantly longer error force release durations (546.6 ± 233.7 ms) than younger adults (318.8 ± 204.1 ms; Welch’s *t* = 2.99, *p* = 0.0055, Hedge’s g = 1.02, 95% CI [0.32, 1.72]), which remained significant after Bonferroni correction (alpha = 0.025).

## 4 Discussion

The present study investigated age-related differences in grip-force regulation using a go/no-go target grip force matching paradigm that quantified reaction times and execution durations for both grip initiation and termination, as well as commission errors and their associated force dynamics. Three principal findings emerged: first, grip initiation was preserved in older adults, as evidenced by comparable grip-onset reaction times and force rise durations across age groups. Second, grip-force termination was selectively slowed with aging, reflected in delayed grip offset reaction times and prolonged release durations. Third, although older adults committed more commission errors on no-go trials, the temporal structure of erroneous responses mirrored that of correct go trials: error-onset reaction times did not differ between groups, whereas error force-release durations were significantly prolonged in older adults. Together, these behavioral results suggest that healthy aging selectively affects processes supporting the termination of ongoing grip-force output, rather than producing a generalized slowing of sensorimotor performance.

### 4.1 Dissociating response selection from execution dynamics

A key contribution of the present work lies in the use of a continuous force go/no-go paradigm that provides separate behavioral indices for the timing of response initiation and termination, as well as the execution dynamics of force generation and release. Conventional go/no-go and stop-signal paradigms typically rely on discrete button presses involving ballistic movements, such that measured “reaction times” combine stimulus detection, response selection, and motor execution in a single latency measure, limiting inferences about how aging affects movement regulation once a response has been initiated (Verbruggen and Logan, 2008; Raud et al., 2020). In contrast, the present target force–matching task required participants to generate and maintain a controlled submaximal grip force, followed by rapid release upon cue offset. This design preserved continuous force output and enabled independent quantification of reaction times associated with grip initiation and termination, as well as the execution dynamics of force generation and release. Using this approach, we found that older adults initiated grip responses comparably to younger adults but exhibited pronounced slowing during grip release. This pattern is consistent with the idea that age-related slowing observed in traditional reaction time tasks may be driven, at least in part, by impairments in terminating or inhibiting ongoing motor outputs rather than by uniform delays in perceptual processing or response selection. Notably, many activities of daily living depend on the regulation and timely release of sustained submaximal grip forces rather than ballistic movements, underscoring the significance of task paradigms that explicitly probe these aspects of motor control when characterizing age-related change.

### 4.2 Selective vulnerability of grip-force release with aging

The most robust age-related differences in this study were confined to grip-force termination. Older adults exhibited both delayed initiation of force release and prolonged release duration, despite preserved grip-onset reaction times and force rise durations. This dissociation argues against a global decline in motor speed and instead points to selective vulnerability of processes required to disengage from an active motor command with typical aging.

Grip force release is not a mirror reverse of grip force generation. Terminating a sustained grip requires inhibition of already recruited motor units, integration of sensory feedback, and coordination of motor state transitions (Johansson, 2002; Johansson, 1998; Johansson and Cole, 1992). Prior work has demonstrated that older adults are disproportionately impaired during the release phase of grip force control compared to force generation in continuous tracking tasks, even when the force capacity is matched across age groups (Davidson et al., 2024). Recent work in similar paradigms further indicates that aging-related force-control deficits are exacerbated when tasks place greater demands on visual feedback and stability of submaximal grip, and that older adults show increased variability and reduced precision in grip-force modulation. For example, Davidson et al (Davidson et al., 2024). showed (in a smaller cohort of 10 young and 10 older adults) that older adults exhibited greater deficits during grip force release than force generation in a force-tracking task; these impairments are linked to both sensory degradation and central changes in control circuitry with aging. The present results align closely with this literature and extend it to a larger cohort by demonstrating selective slowing of release initiation even when perceptual demands and task difficulty are carefully controlled.

Neurophysiological studies further suggest that older adults recruit more widespread cortical resources during grip-force regulation, potentially reflecting compensatory strategies for age-related structural and neurochemical change (Crowell et al., 2020; Mattay et al., 2002; Berger et al., 2020). Importantly, converging evidence indicates that grip-force release engages partially distinct, or differentially weighted functional networks relative to grip force onset, with greater involvement of prefrontal and cingulate control regions implicated in monitoring, inhibition, and switching out of an ongoing motor state (Spraker et al., 2009; Kuhtz-Buschbeck et al., 2001). Functional MRI evidence indicates that force relaxation is supported by a different balance of prefrontal–striatal versus motor cortical–striatal contributions compared to that engaged in force generation (Spraker et al., 2009). Critically, these neural differences were not accounted for by force amplitude, rate, or variability (Spraker et al., 2009). Within this framework, selective slowing of grip-offset reaction time and release duration in aging may reflect vulnerability of control networks involved in terminating an active motor state; in the context of releasing an ongoing grasp., the prefrontal–striatal and anterior cingulate circuitry represent plausible contributors, while force generation mechanisms remain relatively preserved.

Visual inspection of individual data points in Figures 3, 4 suggests greater dispersion in several timing measures among older adults compared with younger adults, consistent with increased inter-individual variability in release-related performance. However, the present sample was not powered to formally model heterogeneity or identify subgroups, and these observations should therefore be interpreted descriptively.

### 4.3 Intracortical inhibition and deficits in grip-force release

Mechanistically, what might underlie the selective slowing of grasp termination but not grasp onset? Neurophysiological studies provide a plausible framework for interpreting the selective slowing of grasp termination observed here, although the present data do not directly measure the neural mechanisms. Relaxing an ongoing grip is an actively regulated process mediated in part by intracortical inhibition (Motawar et al., 2012; Motawar et al., 2016). Transcranial magnetic stimulation (TMS) studies demonstrate that short-interval intracortical inhibition (SICI), a GABA-mediated intracortical inhibitory mechanism, increases during voluntary muscle relaxation (Motawar et al., 2012). This increase in SICI has been shown to be muscle-specific, emerging in the agonist muscle undergoing relaxation (Motawar et al., 2012; Motawar et al., 2016) and, in some cases, extending to synergistic muscles, consistent with coordinated inhibition during grip release (Buccolieri et al., 2004).

Age-related alterations in SICI modulation have been linked to delayed grip relaxation and impaired termination of force output (Motawar et al., 2012; Motawar et al., 2016; Buccolieri et al., 2004). For example, Motawar et al. reported that young adults upregulate SICI during grip relaxation, whereas older adults show diminished SICI modulation and prolonged relaxation times (Motawar et al., 2012; Motawar et al., 2016). These findings, together with evidence of age-related reductions in intracortical inhibition across the adult lifespan (Hehl et al., 2020), support the interpretation that diminished inhibitory control within motor cortical circuits may contribute to release-phase slowing. Importantly, in the present study, intracortical inhibition was inferred from this prior work rather than assessed directly; combined behavioral and neurophysiological studies using similar paradigms will be needed to establish mechanistic links between SICI modulation and grip-force release in aging.

### 4.4 Translational relevance of the go/no-go force-matching paradigm in clinical motor impairment

Evidence from clinical populations underscores the functional relevance of intracortical inhibition for grip control (Prodoehl et al., 2009; Levin et al., 2014). Reduced SICI has been reported in conditions characterized by impaired motor inhibition, including stroke (Ding et al., 2018; Ferreiro de Andrade and Conforto, 2018; Naik et al., 2011), where deficits in grip-force modulation and delayed force release are common (Lang et al., 2009; Seo et al., 2009; Ye et al., 2014; Lindberg et al., 2012). In stroke, impaired hand opening and delayed object release represent persistent functional limitations that are closely linked to disrupted inhibitory control within motor cortical circuits (Ding et al., 2018; Plantin et al., 2022). However, because stroke predominantly affects older adults, it is often difficult to dissociate age-expected slowing of inhibitory motor control from lesion-related disruption of motor networks. Within this context, the present go/no-go target force–matching paradigm offers a practical behavioral framework for probing inhibitory control over grip-force release although direct validation of the present task in clinical cohorts is still required.

Because stroke and frailty predominantly affect older adults, it is often difficult to dissociate age-expected slowing from lesion- or frailty-related disruption of motor networks. Normative aging data from the current go/no-go paradigm may therefore serve as a behavioral reference for future work aimed at identifying release-specific impairments that exceed those expected from healthy aging alone. Similarly, maximal grip strength is widely used as a clinical biomarker of aging and physical frailty and is incorporated into several frailty indices and prognostic models in aging (Vaishya et al., 2024). Reduced grip strength has been associated with adverse outcomes including functional decline, falls, hospitalization, and mortality (do Amaral et al., 2024). However, maximal grip strength represents a static measure of force capacity and provides limited information about the temporal precision, inhibitory control, and sensorimotor coordination required for everyday object manipulation. The present findings suggest that grip-force release dynamics could potentially complement strength-based measures by capturing the ability to disengage an active motor command rapidly; however, their value for frailty screening and prognosis remains to be established in longitudinal and clinical studies.

By quantifying grip-force termination under controlled conditions, the go/no-go target force–matching paradigm extends beyond traditional strength-based assessments. As such, grip-offset dynamics may provide a complementary behavioral marker that better reflects the complexity of age-related motor decline and could be explored in future work as a candidate measure for frailty assessment, particularly in individuals who retain adequate strength but exhibit subtle impairments in motor inhibition and control.

### 4.5 Limitations

Several limitations should be acknowledged. First, the sample size was modest and the study was cross-sectional, which limits inferences about individual aging trajectories and warrants confirmation in larger and longitudinal cohorts. Second, the sample was predominantly female in both age groups; although prior work has not consistently shown sex differences in grip-force timing at submaximal levels, the strong sex imbalance limits generalizability and prevents meaningful examination of sex effects. Third, although the behavioral paradigm provides separate indices of response initiation and force execution, underlying neural mechanisms such as intracortical inhibition were inferred from prior literature rather than measured directly; future work combining TMS or neuroimaging with this task will be necessary to test mechanistic hypotheses. Fourth, the task relied on visually guided force matching, and age-related differences in sensory weighting or visual processing may have influenced performance despite individualized target forces. Finally, participants were healthy community-dwelling older adults, and the present results should be interpreted as preliminary normative behavioral data rather than definitive age benchmarks; generalization to clinical populations, including those with stroke, Parkinson’s disease, or frailty will require direct validation in patient cohorts.

## 5 Conclusion

In summary, the present findings indicate that aging selectively slows grip-force release while sparing grip initiation and force generation. By providing separate behavioral measures of response initiation and execution within a single force-control paradigm, the study suggests that delayed force termination is consistent with difficulty disengaging from an ongoing motor command rather than generalized motor slowing. These behavioral results converge with prior work demonstrating release-specific vulnerability in aging and extend it by characterizing inhibitory-control failures on no-go trials within the same task. Given that impaired hand opening and delayed object release are common and functionally limiting in conditions such as stroke, these normative aging data from this paradigm may provide a useful behavioral reference for future studies aimed at identifying release-specific impairments that exceed those from healthy aging.

## Data Availability

The raw data supporting the conclusions of this article will be made available by the authors, without undue reservation; requests can be directed to geedlab@utep.edu.

## References

[ref1] Berger A. Steinberg F. Thomas F. Doppelmayr M. (2020). Neural correlates of age-related changes in precise grip force regulation: a combined EEG-fNIRS study. Front. Aging Neurosci. 12:594810. doi: 10.3389/fnagi.2020.594810, 33362531 PMC7759198

[ref2] Bohannon R. W. (2019). Grip strength: an indispensable biomarker for older adults. Clin. Interv. Aging 14, 1681–1691. doi: 10.2147/CIA.S194543, 31631989 PMC6778477

[ref3] Buccolieri A. Abbruzzese G. Rothwell J. C. (2004). Relaxation from a voluntary contraction is preceded by increased excitability of motor cortical inhibitory circuits. J. Physiol. 558, 685–695. doi: 10.1113/jphysiol.2004.064774, 15181164 PMC1664966

[ref4] Carvalho C. R. Fernández J. M. Del-Ama A. J. Oliveira Barroso F. Moreno J. C. (2023). Review of electromyography onset detection methods for real-time control of robotic exoskeletons. J. Neuroeng. Rehabil. 20:141. doi: 10.1186/s12984-023-01268-8, 37872633 PMC10594734

[ref5] Crowell C. A. Davis S. W. Beynel L. Deng L. Lakhlani D. Hilbig S. A. . (2020). Older adults benefit from more widespread brain network integration during working memory. NeuroImage 218:116959. doi: 10.1016/j.neuroimage.2020.116959, 32442638 PMC7571507

[ref6] Davidson S. Learman K. Zimmerman E. Rosenfeldt A. B. Koop M. Alberts J. L. (2024). Older adults are impaired in the release of grip force during a force tracking task. Exp. Brain Res. 242, 665–674. doi: 10.1007/s00221-023-06770-y, 38246931 PMC10894767

[ref7] Ding Q. Triggs W. J. Kamath S. M. Patten C. (2018). Short intracortical inhibition during voluntary movement reveals persistent impairment post-stroke. Front. Neurol. 9:1105. doi: 10.3389/fneur.2018.01105, 30662425 PMC6328452

[ref8] Divers R. Ham L. Matchanova A. Hackett K. Mis R. Howard K. . (2021). When and how did you go wrong? Characterizing mild functional difficulties in older adults during an everyday task. Neuropsychol. Dev. Cogn. B Aging Neuropsychol. Cogn. 28, 308–326. doi: 10.1080/13825585.2020.1756210, 32352347 PMC7606330

[ref9] do Amaral C. M. S. S. B. da Luz Goulart C. Silva B. M. Valente J. Rezende A. G. Fernandes E. . (2024). Low handgrip strength is associated with worse functional outcomes in long COVID. Sci. Rep. 14:2049. doi: 10.1038/s41598-024-52401-z, 38267519 PMC10808118

[ref10] Ferreiro de Andrade K. N. Conforto A. B. (2018). Decreased short-interval intracortical inhibition correlates with better pinch strength in patients with stroke and good motor recovery. Brain Stimul. 11, 772–774. doi: 10.1016/j.brs.2018.01.030, 29426671 PMC6019556

[ref11] Goldenkoff E. R. Logue R. N. Brown S. H. Vesia M. (2021). Reduced facilitation of parietal-motor functional connections in older adults. Front. Aging Neurosci. 13:595288. doi: 10.3389/fnagi.2021.595288, 33597858 PMC7882479

[ref12] Hardwick R. M. Forrence A. D. Costello M. G. Zackowski K. Haith A. M. (2022). Age-related increases in reaction time result from slower preparation, not delayed initiation. J. Neurophysiol. 128, 582–592. doi: 10.1152/jn.00072.2022, 35829640 PMC9423772

[ref13] Hehl M. Swinnen S. P. Cuypers K. (2020). Alterations of hand sensorimotor function and cortical motor representations over the adult lifespan. Aging (Albany NY) 12, 4617–4640. doi: 10.18632/aging.102925, 32160591 PMC7093194

[ref14] Johansson R. S. (1998). Sensory input and control of grip. Novartis Found. Symp. 218, 45–59–discussion 59–63. doi: 10.1002/9780470515563.ch4, 9949815

[ref15] Johansson R. (2002). “Dynamic use of tactile afferent signals in control of dexterous manipulation,” in Sensorimotor Control of Movement and Posture, eds. Gandevia S. Proske U. Stuart D., vol. 508 (Boston, MA: Springer US), 397–410. 10.1007/978-1-4615-0713-0_45 12171136

[ref16] Johansson R. S. Cole K. J. (1992). Sensory-motor coordination during grasping and manipulative actions. Curr. Opin. Neurobiol. 2, 815–823. doi: 10.1016/0959-4388(92)90139-C, 1477545

[ref17] Kuhtz-Buschbeck J. P. Ehrsson H. H. Forssberg H. (2001). Human brain activity in the control of fine static precision grip forces: an fMRI study. Eur. J. Neurosci. 14, 382–390. doi: 10.1046/j.0953-816x.2001.01639.x, 11553288

[ref18] Lang C. E. DeJong S. L. Beebe J. A. (2009). Recovery of thumb and finger extension and its relation to grasp performance after stroke. J. Neurophysiol. 102, 451–459. doi: 10.1152/jn.91310.2008, 19458140 PMC2712280

[ref19] Levin O. Fujiyama H. Boisgontier M. P. Swinnen S. P. Summers J. J. (2014). Aging and motor inhibition: a converging perspective provided by brain stimulation and imaging approaches. Neurosci. Biobehav. Rev. 43, 100–117. doi: 10.1016/j.neubiorev.2014.04.001, 24726575

[ref20] Lin B. S. Kuo S. F. Lee I. J. Lu L. H. Chen P. Y. Wang P. C. . (2021). The impact of aging and reaching movements on grip stability control during manual precision tasks. BMC Geriatr. 21:703. doi: 10.1186/s12877-021-02663-3, 34911487 PMC8672550

[ref21] Lin C. H. Sung W. H. Chiang S. L. Lee S. C. Lu L. H. Wang P. C. . (2019). Influence of aging and visual feedback on the stability of hand grip control in elderly adults. Exp. Gerontol. 119, 74–81. doi: 10.1016/j.exger.2019.01.024, 30695717

[ref22] Lindberg P. G. Roche N. Robertson J. Roby-Brami A. Bussel B. Maier M. A. (2012). Affected and unaffected quantitative aspects of grip force control in hemiparetic patients after stroke. Brain Res. 1452, 96–107. doi: 10.1016/j.brainres.2012.03.007, 22464180

[ref23] Massy-Westropp N. M. Gill T. K. Taylor A. W. Bohannon R. W. Hill C. L. (2011). Hand grip strength: age and gender stratified normative data in a population-based study. BMC. Res. Notes 4:127. doi: 10.1186/1756-0500-4-127, 21492469 PMC3101655

[ref24] Mattay V. S. Fera F. Tessitore A. Hariri A. R. das S. Callicott J. H. . (2002). Neurophysiological correlates of age-related changes in human motor function. Neurology 58, 630–635. doi: 10.1212/WNL.58.4.630, 11865144

[ref25] McKniff M. Holmqvist S. Kaplan M. Simone S. M. Tassoni M. B. Mis R. E. . (2025). Subtle inefficiencies in everyday tasks indicate early functional difficulties in older adults: implications for clinical practice and research. Clin. Neuropsychol. 40, 183–202. doi: 10.1080/13854046.2025.2497381, 40304615 PMC12353437

[ref26] Motawar B. Hur P. Stinear J. Seo N. J. (2012). Contribution of intracortical inhibition in voluntary muscle relaxation. Exp. Brain Res. 221, 299–308. doi: 10.1007/s00221-012-3173-x, 22791231 PMC8103219

[ref27] Motawar B. Stinear J. W. Lauer A. W. Ramakrishnan V. Seo N. J. (2016). Delayed grip relaxation and altered modulation of intracortical inhibition with aging. Exp. Brain Res. 234, 985–995. doi: 10.1007/s00221-015-4527-y, 26686531 PMC4786465

[ref28] Naik S. K. Patten C. Lodha N. Coombes S. A. Cauraugh J. H. (2011). Force control deficits in chronic stroke: grip formation and release phases. Exp. Brain Res. 211, 1–15. doi: 10.1007/s00221-011-2637-8, 21448576

[ref29] Pinto B. L. Callaghan J. P. (2023). Movement onset detection methods: a comparison using force plate recordings. J. Appl. Biomech. 39, 118–123. doi: 10.1123/jab.2022-0111, 36913948

[ref30] Plantin J. Godbolt A. K. Pennati G. V. Laurencikas E. Fransson P. Baron J. C. . (2022). Motor inhibition and its contribution to recovery of dexterous hand use after stroke. Brain Commun. 4:fcac241. doi: 10.1093/braincomms/fcac241, 36262369 PMC9562786

[ref31] Prodoehl J. Corcos D. M. Vaillancourt D. E. (2009). Basal ganglia mechanisms underlying precision grip force control. Neurosci. Biobehav. Rev. 33, 900–908. doi: 10.1016/j.neubiorev.2009.03.004, 19428499 PMC2684813

[ref32] Raez M. B. Hussain M. S. Mohd-Yasin F. (2006). Techniques of EMG signal analysis: detection, processing, classification and applications. Biol Proced Online. 8, 11–35. doi: 10.1251/bpo115, 16799694 PMC1455479

[ref33] Raud L. Westerhausen R. Dooley N. Huster R. J. (2020). Differences in unity: the go/no-go and stop signal tasks rely on different mechanisms. NeuroImage 210:116582. doi: 10.1016/j.neuroimage.2020.116582, 31987997

[ref34] Reuter E. M. Voelcker-Rehage C. Vieluf S. Godde B. (2014). Effects of age and expertise on tactile learning in humans. Eur. J. Neurosci. 40, 2589–2599. doi: 10.1111/ejn.12629, 24863287

[ref35] Ruitenberg M. F. L. Cassady K. E. Reuter-Lorenz P. A. Tommerdahl M. Seidler R. D. (2019). Age-related reductions in tactile and motor inhibitory function start early but are independent. Front. Aging Neurosci. 11:193. doi: 10.3389/fnagi.2019.00193, 31417396 PMC6682653

[ref36] Seo N. J. Rymer W. Z. Kamper D. G. (2009). Delays in grip initiation and termination in persons with stroke: effects of arm support and active muscle stretch exercise. J. Neurophysiol. 101, 3108–3115. doi: 10.1152/jn.91108.2008, 19357330

[ref37] Spraker M. B. Corcos D. M. Vaillancourt D. E. (2009). Cortical and subcortical mechanisms for precisely controlled force generation and force relaxation. Cereb. Cortex 19, 2640–2650. doi: 10.1093/cercor/bhp015, 19254959 PMC2758679

[ref38] Vaishya R. Misra A. Vaish A. Ursino N. D’Ambrosi R. (2024). Hand grip strength as a proposed new vital sign of health: a narrative review of evidences. J. Health Popul. Nutr. 43:7. doi: 10.1186/s41043-024-00500-y, 38195493 PMC10777545

[ref39] Verbruggen F. Logan G. D. (2008). Response inhibition in the stop-signal paradigm. Trends Cogn. Sci. 12, 418–424. doi: 10.1016/j.tics.2008.07.005, 18799345 PMC2709177

[ref40] Vieluf S. Godde B. Reuter E. M. Temprado J. J. Voelcker-Rehage C. (2015). Practice effects in bimanual force control: does age matter? J. Mot. Behav. 47, 57–72. doi: 10.1080/00222895.2014.981499, 25575223

[ref41] Voelcker-Rehage C. Alberts J. L. (2005). Age-related changes in grasping force modulation. Exp. Brain Res. 166, 61–70. doi: 10.1007/s00221-005-2342-6, 16096780

[ref42] Ye Y. Ma L. Yan T. Liu H. Wei X. Song R. (2014). Kinetic measurements of hand motor impairments after mild to moderate stroke using grip control tasks. J. Neuroeng. Rehabil. 11:84. doi: 10.1186/1743-0003-11-84, 24886085 PMC4038706

[ref43] Zammit A. R. Robitaille A. Piccinin A. M. Muniz-Terrera G. Hofer S. M. (2019). Associations between aging-related changes in grip strength and cognitive function in older adults: a systematic review. J. Gerontol. A Biol. Sci. Med. Sci. 74, 519–527. doi: 10.1093/gerona/gly046, 29528368 PMC6417444

